# In Vitro Characterization of an Anodized Surface of a Dental Implant Collar and Dental Abutment on Peri-Implant Cellular Response

**DOI:** 10.3390/ma16176012

**Published:** 2023-09-01

**Authors:** Valeria Traver-Méndez, Octavi Camps-Font, Francesc Ventura, Miquel Angel Nicolau-Sansó, Carles Subirà-Pifarré, Rui Figueiredo, Eduard Valmaseda-Castellón

**Affiliations:** 1 Oral Surgery and Implantology, Faculty of Medicine and Health Sciences, University of Barcelona, 08907 Barcelona, Spain; vtraver7@gmail.com (V.T.-M.); ruibarbosa@ub.edu (R.F.); eduardvalmaseda@ub.edu (E.V.-C.); 2 IDIBELL Institute, 08907 L’Hospitalet de Llobregat, Spain; csubira@ub.edu; 3 Department of Physiological Sciences, Faculty of Medicine and Health Sciences, University of Barcelona, 08907 Barcelona, Spain; fventura@ub.edu; 4 Adult Comprehensive Dentistry, Faculty of Medicine and Health Sciences, University of Barcelona, 08907 Barcelona, Spain; miquelangel15@gmail.com

**Keywords:** dental implants, anodization, surface properties, mesenchymal stem cells, HaCaT keratinocytes

## Abstract

The purpose of this paper was to determine the effect of anodization on the in vitro proliferation and adhesion of immortalized human keratinocytes (HaCats) and mouse bone marrow-derived mesenchymal stem cells (BM-MSCs) in Titanium Grade 23 (Ti6Al4V ELI) discs and to describe the surface topography, roughness, and composition of dental implants (body and collar) and abutments submitted to an area-specific anodization process. HaCat cells and BM-MSCs were seeded onto discs with three different surface treatments: machined, area-specific anodization for abutments, and area-specific anodization for implant collars. Cell proliferation was assessed using a resazurin-based fluorescent dye on days 1, 3, and 7, while cell adhesion was examined using scanning electron microscopy (SEM). Surface topography, roughness, and composition were evaluated for six implant bodies with an anodized rough surface, six anodized implant smooth collars, and six anodized prosthetic abutments. Both HaCats and BM-MSCs showed increased viability over time (*p* < 0.001) with no statistically significant differences among the different surfaces (*p* = 0.447 HaCats and *p* = 0.631 BM-MSCs). SEM analysis revealed an enhanced presence and adhesion of HaCat cells on the anodized surface for the implant collars and an increased adhesion of BM-MSCs on both the anodized and machined surface abutments. The topography characteristics of the treated implants and abutments varied depending on the specific implant region. Chemical analysis confirmed the presence of oxygen, calcium, phosphorus, and sodium on the anodized surfaces. The area-specific anodization process can be utilized to create variable topography, increase the specific surface area, and introduce oxygen, calcium, phosphorus, and sodium to dental implants and abutments. While BM-MSCs and HaCat cells showed similar adhesion and proliferation on anodized and machined surfaces, a positive interaction between anodized Ti6Al4V ELI surfaces and these two cell lines present in the peri-implant mucosa was observed. Due to the limitations of the present study, further research is necessary to confirm these findings.

## 1. Introduction

Dental extractions are performed daily in clinical practices worldwide to replace missing teeth and restore function and esthetics. Among the therapeutic options available, dental implants have gained significant popularity due to their high success rate and predictability [1,2].

In the early 1960s, Brånemark and his collaborators discovered a permanent incorporation of titanium chambers into the bone while conducting a microcirculation study in rabbit fibulae. Subsequently, years later, Dr. Brånemark and his team defined this phenomenon as osseointegration and described it as a “direct structural and functional connection between ordered living bone and the surface of a load-carrying implant” [3]. Since then, dental implant designs have continuously evolved to enhance their biocompatibility with peri-implant tissues and improve biomechanics and esthetics [4,5]. The surface characteristics of dental implants and their abutments play a crucial role in modulating cell responses and facilitating their integration with the bone and soft tissues. Factors such as surface topography, chemical composition, charge, oxide layer thickness, and wettability influence the interaction between the implant and the surrounding tissues [6,7]. 

A wide array of surface modifications has been developed and implemented on commercially available implants using various subtracting and additive methods. These methods include anodic oxidation, sandblasting, acid etching with mineral acids, calcium-phosphate coatings, ultraviolet light treatment, plasma of argon treatment, and combinations of these techniques [8,9]. Notably, Carossa et al. conducted a systematic review of the available evidence on the cellular activity of titanium treated with plasma of argon and reported a potential enhancement of cell adhesion and protein absorption during the initial hours after cell seeding. However, it was also pointed out that the duration of this effect in in vitro studies remains uncertain [9]. 

Electrochemical anodization is one of the techniques used to modify the physicochemical elements of an implant surface. This electrochemical oxidative process has been used to modify certain areas of the implant, changing its surface, porosity, nanostructure, chemical composition, energy, and color [10,11,12,13,14]. Anodization is particularly useful in areas with close contact with the mucosa, as the anodized surface interacts not only with osteoblasts but also with fibroblasts and epithelial cells, potentially reducing the risk of bacterial colonization around the implant [15]. In addition, the anodization process allows for titanium to showcase different colors, effectively masking its inherent grayish hue. This esthetic improvement is especially valuable in cases where we encounter thin mucosa or soft tissue dehiscences [16,17,18].

Peri-implantitis stands out as one of the primary causes of dental implant failure. Establishing an effective mucosal seal is desirable to reduce the risk of these biological complications, as it minimizes submucosal biofilm accumulation [19]. To evaluate this mucosal attachment to the implant collar and dental abutment, several in vitro studies have been conducted, utilizing different primary cells, such as gingival fibroblasts and fibroblastic progenitor cells (mesenchymal stem cells), for the connective tissue, and keratinocytes and epithelial progenitor cells for the epithelial anchorage [20,21,22,23,24].

While implants with anodized collars are currently available on the market and are utilized by clinicians, there is still a scarcity of data regarding their benefits in achieving both esthetics and a mucosal seal. Therefore, the objective of the present study was to compare the in vitro proliferation and adhesion of immortalized human keratinocytes (HaCats) and mouse bone marrow-derived mesenchymal stem cells (BM-MSCs) on anodized titanium (Ti) surfaces. Additionally, this study aimed to describe the surface topography, roughness, and composition of these implants and abutments. 

The hypothesis of the present study proposed that there would be an increased adhesion and proliferation of BM-MSCs and HaCat cells when interacting with an anodized surface compared to machined surfaces.

## 2. Materials and Methods

This in vitro study was conducted in accordance with the Principles of Good Laboratory Practice. 

### 2.1. Materials

Six tapered, screw-shaped Ti6Al4V ELI (Titanium Grade 23) commercial dental implants (n = 6) with a Biomimetic Advanced Surface (BAS) treatment (Avinent^®^ Biomimetic Iceberg implants; Avinent Implant System, Santpedor, Spain) were used to characterize the surface of different regions of the implant. This specific surface treatment is obtained by a combination of two processes: shot blasting procedure and electrochemical treatment with Ca- and P-rich electrolyte solution. (Table 1). The platform and body diameters of the implants were 3.5 mm, and the total body length was 10 mm. The implant threads were V-shaped and measured 0.08 and 0.28 mm in depth at the neck and body of the fixture, respectively.

Additionally, six (n = 6) anodized commercial dental abutments (Avinent^®^ CC.I Healing Abutment, Barcelona, Santpedor, Spain) were used (Table 1) along with six machined abutments and implants as controls (n = 6) (Figure S1).

Furthermore, a total of 42 discs (n = 42) made of Titanium Grade 23 (Ti6Al4V ELI), were used for the in vitro cell proliferation and adhesion assay. These discs with a diameter of 10 mm and a thickness of 2 mm were produced by the manufacturer (Avinent Implant System, Santpedor, Spain). Prior to use, all discs were degreased by washing them with a modified alcohol solution. 

Subsequently, 14 titanium discs (n = 14) were subjected to anodization using the same technique employed for dental abutments (AA group). They underwent a 4 min acid pickling treatment followed by anodization in an acidic electrolyte solution to recreate the golden surface of an abutment. 

The second study group (AC group) consisted of 14 titanium discs (n = 14) that underwent anodic oxidation in a basic Ca- and P-rich electrolyte solution to obtain an identical surface to that of the above-mentioned implant collar.

As for the control group, 14 titanium machined discs (n = 14) were used (MAC group).

All procedures were carried out at room temperature. Finally, all discs were ultrasonically washed with neutral detergent diluted in distilled water, dried, and then steam sterilized (Figure S1).

### 2.2. Scanning Electron Microscopy (SEM)

The surfaces of 6 implants (n = 6) and dental abutments (n = 6) were equally distributed into 2 groups (treated and machined) and were subsequently assessed via SEM (Jeol JSM-7001F, JEOL Ltd., Tokyo, Japan) with a secondary electron detector and a 20 kV acceleration voltage at ×500 and ×1500 magnifications.

SEM images were obtained with a magnification of ×1500 using the ImageJ software 1.53t (National Institutes of Health, Bethesda, MD, USA) to determine their surface quality semi-quantitatively, as well as the presence of impurities, fractures, deformation, scuffing, cracks, or fissures.

### 2.3. Surface Topography Analyses

The remaining 6 implants (n = 6) and 6 dental abutments (n = 6), divided into 2 groups (3 treated and 3 machined), were analyzed with a confocal optical microscope (Plμ2300, Sensofar, Terrassa, Spain) under 500× magnification and a numerical aperture of 0.50. A total of 4 regions of interest of 636 × 442 µm were selected: implant anodized collar (C), neck microthreads (MT), valley (V), and on the tip of a thread (T). The SensoMap Plus 5.1.1 software (Sensofar, Terrassa, Spain) was used to measure the surface topography and calculate the surface roughness parameters.

The surface roughness of each area was defined using the following normalized three-dimensional parameters:

- Sa (arithmetic mean height) is defined as the difference in height of each point compared to the arithmetical mean of the surface. Expressed as µm.
- Sz (average maximum height) is defined as the sum of the largest peak height value and the largest pit depth value within the defined area. Expressed as µm.
- Ssk (skewness of topography height distribution) is defined as the degree of bias of the rough shape.
- Sdr (developed interfacial area ratio) is defined as the ratio between the area of the “real” developed surface and the area of the “projected” surface. Expressed as percentage.

Form was previously removed, and a Gaussian filter of 80 μm for C and V and 25 µm for MT and T was applied for roughness and waviness. Only roughness parameters were assessed.

### 2.4. Surface Chemistry

To determine the composition of the surfaces, energy dispersive X-ray spectroscopy (EDS) was performed on the samples previously examined with SEM using a Jeol JSM-7001F microscope (with EDS elemental analyzer, JEOL Ltd., Tokyo, Japan). The samples were assessed with an electron beam voltage of 15 kV and a beam current of less than 3 × 10^−7^ A. Differential spectra were collected in two areas for each sample to ensure accurate measurements. Atomic concentrations were determined using the relative sensitivity factors for 15 kV, and concentration ratios were calculated using peak height intensities corrected by the appropriate relative sensitivity factors. 

### 2.5. Cellular Assays

HaCats (CLS Cell Lines Services GmbH, Heidelberg, Germany) and BM-MSCs (Physiological Sciences Laboratory, Faculty of Medicine and Health Sciences, University of Barcelona, Spain) were cultured and counted using a Neubauer counting chamber. A medium with 2 × 10^5^/mL HaCat cells and another with 2 × 10^5^/mL BM-MSCs was obtained (T_0_). A total of 36 Ti6Al4V ELI discs (n = 36) were placed inside 3 separate 12-well plates: 150 μL of HaCat cell medium was seeded on top of 18 discs (n = 18), having 6 discs per surface (n = 6), and 150 μL of BM-MSC medium was seeded on top of a different set of 18 discs (n = 18), also equally divided into 6 discs (n = 6) per surface. The remaining 6 discs (n = 6), with 2 of them for each surface (n = 2), were used as controls and submerged in 1 mL of Dulbecco’s Modified Eagle Medium (Figure S1).

### 2.6. Cell Proliferation Analysis

Fifteen Ti6Al4V ELI discs for each cell line, meaning five per surface treatment and cell line, were used for the proliferation analysis (Figure S1). After incubation for the indicated days, cells were collected from each disc and placed on microwells on a 96-well plate. The samples were dyed with 10% of alamarBlue Cell Viability Reagent (Molecular Probes DAL1025, Thermo Fisher Scientific, Waltham, MA, USA) and incubated for 4 h. Fluorescence was then determined using a microplate reader (Sunrise-XFLUOR4 V4.51, TECAN, Mendendorf, Switzerland) at a wavelength of 570 nm and 600 nm. A total of 2 consecutive readings per well were performed on days 1, 3, and 7 after seeding. The proliferation rate between each timepoint and T_0_ was calculated as the percentual increase between them.

### 2.7. SEM of Cell Seeding on Implants

For each surface, 1 HaCat and 1 BM-MSC disc (n = 6) were used for the microscopic analysis on day 1 after seeding to assess cell adhesion (Figure S1). Samples were fixed with glutaraldehyde 2.5% in 0.1 M phosphate buffer, pH 7.4, post-fixed in osmium tetroxide (1%) in the same phosphate buffer, dehydrated in graded alcohol, and processed for critical point drying using Emitech K850. Samples were covered with a carbon thin film in order to improve their electrical conductivity. 

The samples were observed, and images were obtained using a JEOL J7001F SEM (JEOL Ltd., Tokyo, Japan) with a 15 kV acceleration voltage under various magnifications.

### 2.8. Statistical Analysis

To compare the 3 different surface treatments (i.e., MAC, AA, and AC), the sample size was established at 15 discs per group to obtain 80% of statistical power to detect, by means of a one-way ANOVA F-test for independent groups and fixing the significance level to 5%, a Cohen’s f statistic of 1.

Normality of scale variables was explored through Shapiro–Wilk’s test and visual analysis of the P-P and box plots. Since the normality assumption was violated, descriptive statistics involving the median and inter-quartile range (IQR) were employed to provide a descriptive overview of the data. Differences were explored using Mann–Whitney U and Kruskal–Wallis H-tests.

To analyze the influence of the surface variable over the time evolution of HaCat and BM-MSC proliferation rate, a Friedman test was performed. Wilcoxon signed-rank tests on the different combinations of related groups were carried out to examine where the differences occurred. 

The statistical analysis was carried out with Stata14 (StataCorp^®^, College Station, TX, USA). The level of significance was set at *p* < 0.05 using Bonferroni’s correction for multiplicity of contrasts.

## 3. Results

### 3.1. Scanning Electron Microscopy

**Figure 1:**
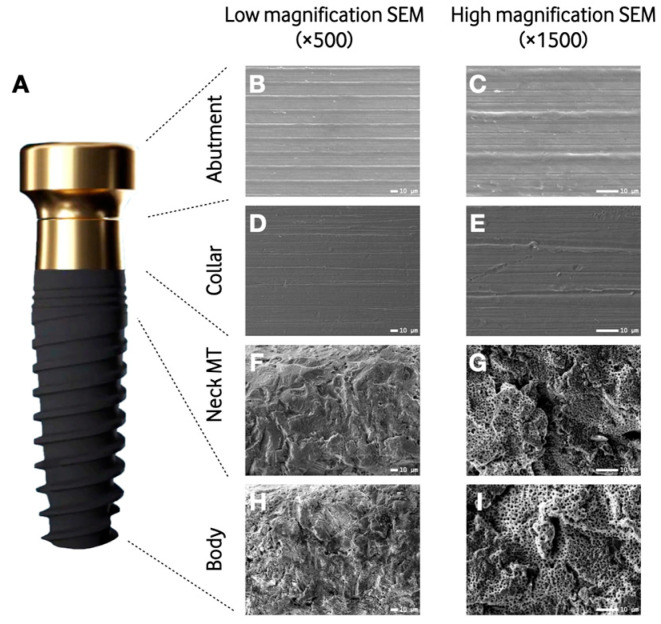
Computer-assisted representation of the implant system depicting the different engineered surfaces (**A**). Microscopic characterization of the four regions of the implant system: abutment (**B**,**C**), implant collar (**D**,**E**), neck microthreads (**F**,**G**), and body (**H**,**I**). Low- (**B**,**D**,**F**,**H**) and high-magnification scanning electron micrographs of the regions of the implant system (**C**,**E**,**G**,**I**). SEM: Scanning electron microscopy; MT: Microthreads.

The SEM images revealed a variable topography of the implants’ and dental abutments’ surfaces according to their location. The topography of group AA showed a non-porous surface (Figure 1B,C). The surface of the implant collar (0–1.8 mm) of the group AC yielded a relatively homogenous surface with the presence of turning lines and pits (Figure 1D,E). The implant body showed a microrough surface due to the sandblasting process with the presence of pores evenly distributed over the surface achieved during the incorporation of Ca and P in the electrochemical treatment (Figure 1F–I).

Low-magnification (×500) SEM photomicrographs of the control samples exhibited a relatively homogenous turned surface. Higher magnification micrographs (×1500) revealed irregular striations, which were caused by the machining process itself.

### 3.2. Surface Topography Analysis

Table 2 shows the values of the different microtopographic parameters used to quantify the surface roughness. The implant body areas of the treated samples were associated with a higher roughness than the machined ones (*p* < 0.001). The increase in the S_a_ parameter was mainly attributed to the plastic deformation produced by the impact of particles on the surface during the sandblasting process. Anodization allowed for an increase in the specific surface of the material, creating a microporosity, but its impact on the Sa value was lower than the sandblasting process.

### 3.3. Surface Composition

The chemical analysis revealed the presence of oxygen, calcium, and phosphorus on the treated samples with the BAS surface (Table 3). These elements were introduced onto the surface during the anodization process.

### 3.4. Cellular Assays

#### 3.4.1. Cell Proliferation Analysis

All cell lines proliferated on the three surfaces. Both HaCats and BM-MSCs significantly increased in number with time (*p* < 0.001), with no differences between surfaces (*p* = 0.447 for HaCats and *p* = 0.631 for BM-MSCs). In addition, the proliferation pattern of HaCats and BM-MSCs was similar on each of the three assessed surfaces (*p* = 0.725 for HaCats and *p* = 0.731 for BM-MSCs). Supplementary Table S1 depicts the cell count for each cell line, surface, and timepoint.

**Figure 2:**
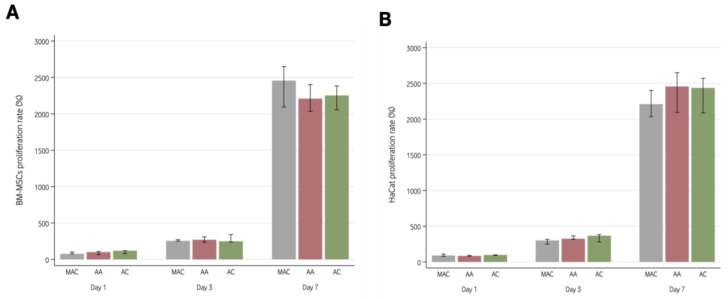
Proliferation rate of HaCats (**A**) and BM-MSCs (**B**) for the three groups: machined (MAC), anodization for dental abutments (AA), and anodization for implant collar (AC). Data expressed as median and interquartile range. BM-MSCs: Mouse bone marrow-derived mesenchymal stem cells; HaCats: Immortalized human keratinocytes.

Figure 2 shows the proliferation rate of both cell lines on the 3 Ti6Al4V ELI surfaces considering the fluorescence readings obtained at day 1, 3, and 7. The results showed an increased number of cells throughout time (*p* < 0.001) with no statistically significant differences between the different surfaces evaluated (*p* = 0.447 for HaCats and *p* = 0.631 for BM-MSCs). 

#### 3.4.2. SEM of Cell Seeding on Implants

**Figure 3:**
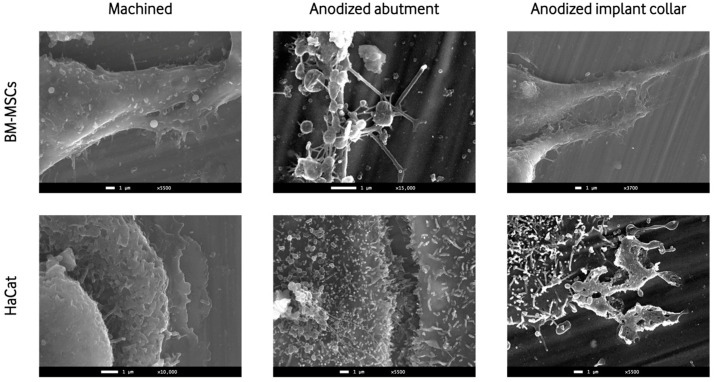
Representative scanning electron micrographs taken under various magnifications showing BM-MSCs and HaCats adhered to the 3 different surfaces on day 1. BM-MSCs: Mouse bone marrow-derived mesenchymal stem cells; HaCats: Immortalized human keratinocytes.

The SEM images showed the presence and adhesion of BM-MSCs and HaCat cells on both anodized surfaces and machined surfaces. Multiple images at various magnifications showed cell-to-cell junctions and cellular filopodia extensions, showing the adhesion and viability of both cellular lines to the titanium discs (Figure 3).

## 4. Discussion

The present research assessed the interaction of peri-implant tissue cells (keratinocytes and mesenchymal cells) with anodized surfaces in an “in vitro” setting. We found similar cell proliferation and adhesion features in both the anodized and the machined surfaces; thus, our hypothesis was rejected. Nevertheless, these results should be treated with caution given that the seeding of the cells was not performed on actual implants but on titanium discs mimicking the implant’s surfaces (collar and abutment). 

The main aim of surface treatments in implant dentistry is to favor and enhance tissue integration to increase success rates and reduce treatment times [15]. Cell interaction with the surface is a key factor in this process [25]. In previous years, the studies that were conducted used to focus on the proliferation and adhesion of bone cells, suggesting that the success of implant treatments relied mainly on the interaction between the implant’s surface and these cells [26,27]. Such is the case of Schwartz et al. who observed an increased proliferation of osteoblasts when titanium rough surfaces were involved [26]. In the same manner, Albrektsson et al. concluded that moderately rough surfaces achieved stronger bone responses than smoother or rougher surfaces and discussed the possible advantages that bioactive implants could offer [27].

Other studies have also stated that a crucial element to determine the success or failure of an implant treatment in both the short- and long-term is the implant’s collar and its micro- and macrostructure [28,29]. The reason for this relies mainly on the proven intimate relation between this component and marginal bone loss [28]. Indeed, Randall et al. [29] compared the clinical and radiographic outcomes of dental implants with roughened collars with and without microgrooves. After 1 year of follow-up, the microgroove-roughened collar surfaces had significantly lower radiographic marginal bone loss, showing that the collar surface seems to influence cell interaction in the crestal area. Likewise, Sul et al. [10] performed a study comparing titanium implants with two different oxide layers (17–200 nm vs. 600–1000 nm) and concluded that a wider oxide layer provided a higher quantity of newly formed bone around the implant, higher bone to implant contact (BIC), and a more pronounced osteoconductive response. Other research highlights not only the importance of marginal bone for the success of implant treatments but also the importance of the formation of a soft-tissue cuff of epithelial cells and fibroblasts, especially in the abutment area [8,30]. Taking this into account, we chose to analyze BM-MSCs because of their capacity to differentiate into bone cells or fibroblasts [31]. The adhesion and proliferation of this type of cell in the implant collar could indicate a better integration of bone and connective tissue, which is also found in this area. In addition to BM-MSCs, keratinocytes were also studied because of their key participation in the formation of the already-mentioned mucosal seal [30,32]. Our results showed that all tested surfaces had good results in terms of adhesion and proliferation of both cell lines, so they seem to be adequate for clinical use. These results are in line with those reported by Gould et al. [33] since they stated that epithelial cells formed an attachment to titanium surfaces by means of hemi-desmosomes. Likewise, Musano et al. performed an in vitro study to analyze the interaction of keratinocytes and fibroblasts with anodized and machined surfaces, finding that there is a favorable adhesion and viability of both cell lines in both surfaces. Although, they state that anodized surfaces increased adhesion for both cell lines at 10 min and enhanced their viability at 2 and 3 days [15]. These results differ from the ones obtained in our study, as we did not find statistically significant differences in terms of adhesion and proliferation when comparing anodized and machined surfaces. Nevertheless, they also state that this response was not maintained when observing cell morphology and focal adhesion, especially with HaCat cells [15]. On the other hand, the results by Dorkhan et al. seem to corroborate our findings, as they state that they did not find statistically significant differences in the adhesion and viability of keratinocytes when comparing commercially pure titanium and two anodized surfaces [34].

The effect of different abutment surface treatments on peri-implant tissues has been widely investigated, but the results from in vivo studies still seem controversial. Pera et al. performed a multi-center, split-mouth, randomized control trial comparing the effect on peri-implant tissue of abutments with chromium nitride/ niobium nitride coatings (superlattice) and machined surfaces, finding no differences between the two after a six-month observational period [35]. On the contrary, other studies found that the anodized transmucosal components, which present minimal roughness (Sa of 0.5–1.0 μm), seem to show a lower marginal bone loss than smoother surfaces [36,37]. Wang et al. [38] stated that the anodization process improves the wettability of the surface, resulting in higher cell adhesion, namely gingival fibroblasts. Nevertheless, just as in our research, anodized surfaces did not produce a significant improvement in cell viability. Milleret et al. [12] tested four different anodized surfaces: abutment, implant collar, transition zone, and implant apex. Each area presented a different oxide layer width, and the response of human gingival keratinocytes and bone marrow-derived MSCs were evaluated. Histocompatibility and topographic studies showed that a better epithelial and fibroblast attachment was possible through the anodization of the implant collar or abutments since it increases the oxide layer thickness and enlarges the surface [12]. These findings suggest that anodized abutments and implant collars may favor peri-implant epithelial tissue insertion. In this regard, our SEM images showed a correct adhesion of the two studied cell lines in all titanium disc surfaces, which confirms previous findings [39,40].

The images obtained via SEM showed a variable surface topography of the treated implants and abutments. The abutment showed a non-porous surface, the collar of the implant showed a relatively homogenous surface with the presence of turning lines and pits, and the implant body showed a microrough surface. These results are like the ones described in the in vitro study by Milleret et al. [12]. The smooth surface found in the treated abutments and the mildly rough surface of the collar could favor fibroblast and epithelial cells’ attachment, allowing for an effective mucosal seal and adequate plaque control [15,41,42]. In our study, the implant body areas of the treated samples were associated with a higher roughness than the machined ones, which according to other investigations, could favor the adhesion of osteogenic cells, promoting osseointegration [43]. Regarding the composition, anodized surfaces exhibit more hydroxyl groups in comparison to sand-blasted and acid-etched implants [44]. Also, as found through the energy dispersive X-ray spectroscopy, during anodization, oxygen, calcium, phosphorus, and sodium are incorporated into the surfaces. These findings have been reported previously [12] and could improve the biological properties of the surface when it comes to cell interaction and attachment [45].

Another clinically relevant factor in the treated surfaces is the color alteration obtained after the anodic oxidation. This could be especially favorable when a thin biotype is present or in cases of soft or bone tissue defects [38,46,47,48,49]. Indeed, it is common to see patients with implant-supported restorations in the anterior area of the maxilla with a greyish color in the peri-implant mucosa that seriously affects the esthetic outcome of the treatment.

The present study has some limitations that need to be considered. Firstly, the use of an “in vitro” design based on a static cell method might not be totally extrapolated to a clinical setting. To provide a more comprehensive understanding, future research should consider incorporating other cell lines or techniques, such as immunostaining or gene expression analysis via qPCR, to assess cellular responses in a more relevant and dynamic environment closely resembling oral conditions. Additionally, to better evaluate cell adhesion and depict the evolution of this dynamic process, it is advisable to reduce the time intervals between observations during experimentation. Future studies should consider the advantages of assessing the cell adhesion in the first 2, 4, and 6 h after seeding. Such an approach, as suggested by some studies, could offer valuable insights into the initial stages of cell–substrate interactions, including the initial attachment, spreading, and focal adhesion [50].

Moreover, while our study examined the impact of anodization on surface roughness and color alteration, it is crucial to recognize that these modifications can have broader implications on the clinical performance of implants. Therefore, it is necessary to perform additional randomized clinical studies (RCT) with a long follow-up to compare the clinical, biological, and esthetic outcomes of anodized and standard surfaces.

## 5. Conclusions

Anodization can be performed in specific implant and dental abutment regions, changing the topography and adding oxygen, calcium, phosphorus, and sodium to the surfaces. Despite this process not showing a significant increase in BM-MSCs and HaCat cell adhesion and proliferation compared to machined surfaces, it is essential to highlight that our study does confirm a positive interaction between keratinocytes and BM-MSCs with anodized Ti6Al4V ELI surfaces. This finding further supports the potential utility of such surfaces in dental implant collars and abutments, which could offer a positive interaction with the cell lines present in the peri-implant mucosa.

Due to the limitations of the present in vitro study, further research is required to confirm these findings and to fully understand the possible advantages of using anodized surfaces in dental implants and abutments.

## Figures and Tables

**Table 1:** Anodization parameters of the different implant sites: voltage (volts), time (seconds), and calcium–phosphorous ratio.

	Voltage	Time	Ratio Ca-P
Implant collar	58 V	90 s	2:1
Implant body	138 V	90 s	2:1
Prosthetic abutment	58.8 V	75 s	0

**Table 2:** Results of the different microtopographic parameters used to quantify the surface roughness. Data expressed as median and interquartile range.

	Sa (µm)	Sz (µm)	Ssk	Sdr (%)
**Treated samples (n = 3)**				
Prosthetic abutment	0.25 (0.06)	1.19 (0.55)	−0.05 (0.12)	0.43 (0.72)
Implant collar	0.11 (0.02)	1.28 (0.57)	−0.02 (0.27)	0.62 (0.83)
Implant neck microthreads	1.20 (0.11)	18.37 (1.87)	−0.20 (0.30)	74.52 (21.73)
Implant body thread	0.95 (0.58)	18.58 (8.93)	−0.27 (0.31)	61.38 (20.52)
Implant body valley	1.68 (0.24)	16.38 (1.67)	−0.12 (0.27)	56.38 (18.57)
**Machined samples (n = 3)**				
Prosthetic abutment	0.13 (0.02)	1.11 (0.68)	−0.05 (0.26)	0.28 (0.21)
Implant collar	0.10 (0.03)	1.08 (0.71)	0.02 (0.23)	0.23 (0.24)
Implant neck microthreads	0.13 (0.07)	1.79 (2.21)	−0.20 (0.79)	1.12 (0.32)
Implant body thread	0.07 (0.08)	2.21 (0.76)	0.79 (4.65)	0.32 (0.85)
Implant body valley	0.14 (0.03)	1.44 (0.65)	0.22 (0.27)	0.55 (0.04)

**Table 3:** Weight percentage (%) of chemical composition in the different samples analyzed. Data expressed as median (interquartile range).

	O	Na	Al	P	Ca	Ti	V
**Treated samples (n = 3)**							
Prosthetic abutment	30.15 (1.96)	-	4.17 (0.32)	0.29 (0.09)	-	62.71 (2.17)	2.37 (0.21)
Implant collar	31.52 (0.85)	-	4.53 (0.20)	-	-	61.82 (0.55)	2.22 (0.58)
Implant Body	46.44 (1.04)	0.44 (0.13)	9.24 (0.30)	1.05 (0.31)	0.63 (0.26)	41.22 (1.37)	1.67 (0.13)
**Machined samples (n = 3)**							
Prosthetic abutment	-	-	6.42 (0.31)	-	-	90.15 (0.28)	3.23 (0.20)
Implant collar	-	-	6.61 (0.37)	-	-	90.45 (0.26)	3.41 (0.44)
Implant Body	-	-	5.95 (0.66)	-	-	90.17 (0.30)	3.60 (0.38)

O: Oxygen; Na: Sodium; Al: Aluminum; P: Phosphate; Ca: Calcium; Ti: Titanium; V: Vanadium.

## Data Availability

Not applicable.

## Supplementary Materials

The following supporting information can be downloaded at: https://www.mdpi.com/article/10.3390/ma16176012/s1, Figure S1: Distribution of the Ti6Al4V ELI discs into groups according to the type of surface, cell-line seeded, and the analysis performed. Table S1: Fluorescence readings showing the proliferation of HaCat and BM-MSCs on the machined surface (MAC), the surface with anodization for abutments (AA) and the anodization for implant collar (AC), at day 1, 3 and 7. Data expressed as median (interquartile range).
