# Supplementary material for: In Vitro Characterization of an Anodized Surface of a Dental Implant Collar and Dental Abutment on Peri-Implant Cellular Response
Source: Materials (Basel). 2023 Sep 1;16(17):6012. doi: 10.3390/ma16176012 (PMC10489139; doi:10.3390/ma16176012)
Supplement: Supplementary file 1 [file materials-16-06012-s001.zip › materials-2513183-supplementary.pdf]

materials

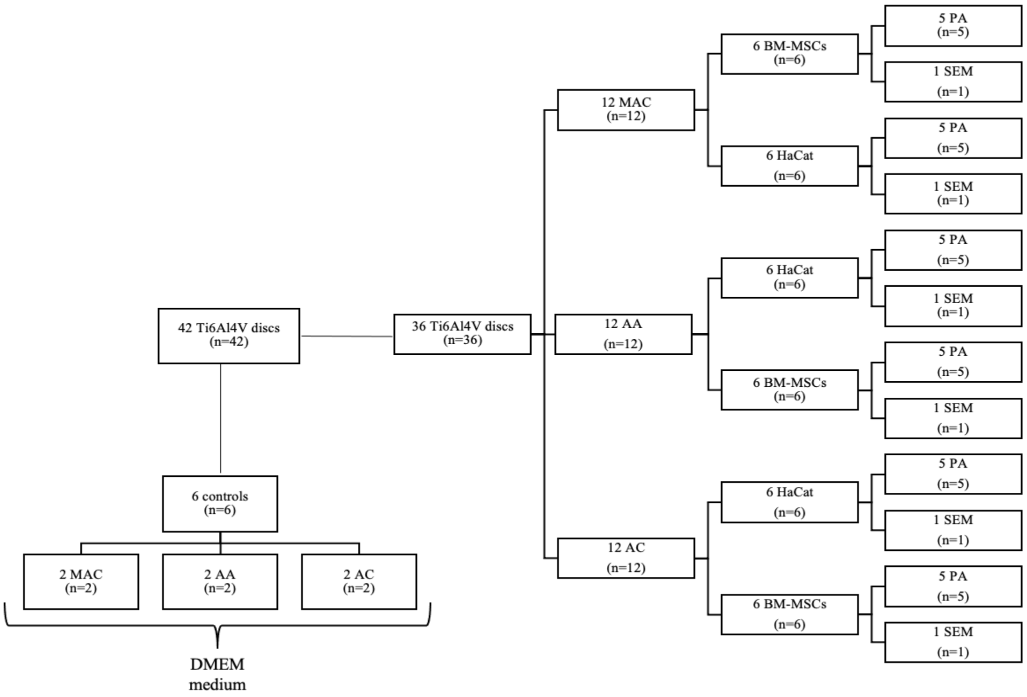

Figure S1. Distribution of the Ti6Al4V ELI discs into groups according to the type of surface, cell-line seeded, and the analysis performed.
MAC: Machined surface; AA: Anodization for dental abutments; AC: Anodization for implant collar; DMEM: Dulbecco's Modified Eagle Medium; BM-MSCs: Mouse bone marrow derived mesenchymal stem-cells; HaCat: Immortalized human keratinocytes; PA: Proliferation Analysis; SEM: Scanning Electron Microscopy.

Supplementary Table S1. Fluorescence readings showing the proliferation of HaCat and BM-MSCs on the machined surface (MAC), the surface with anodization for abutments (AA) and the anodization for implant collar (AC), at day 1, 3 and 7. Data expressed as median (interquartile range).

	**HaCat**			
	MAC	AA	AC	P-value
Day 1	1810 (621.23)	1683.43 (159.59)	1947.45 (139.63)	0.566
Day 3	6005.86 (1329.01)	6499.10 (972.78)	7328.02 (2041.47)	0.605
Day 7	44181.22 (7400.69)	49137.65 (11134.98)	48730.27 (9709.16)	0.454
	**BM-MSCs**			
	MAC	AA	AC	P-value
Day 1	1528.86 (555.58)	2018.91 (875.64)	2387.79 (808.84)	0.756
Day 3	5101.59 (424.74)	5409.86 (1466.02)	4985.13 (2041.47)	0.961
Day 7	49137.65 (11134.98)	44181.22 (7400.69)	45063.87 (6518.04)	0.651
